# Large Platform Growth Effect of Single-Crystal Diamond on the Regulation of Its Dielectric Properties and Stress for THz Applications

**DOI:** 10.3390/ma18204745

**Published:** 2025-10-16

**Authors:** Pengwei Zhang, Jun Zhou, Hui Song, Chenxi Liu, He Li, Guoyong Yang, Peng Sun, Yiming Nan, Jian Yi, Huiping Bai, Yuezhong Wang, Nan Jiang, Kazuhito Nishimura

**Affiliations:** 1 Key Laboratory of Advanced Marine Materials, Ningbo Institute of Materials Technology and Engineering, Chinese Academy of Sciences, Ningbo 315201, China; zhangpengwei@nimte.ac.cn (P.Z.);; 2 China School of Materials and Energy, Yunnan University, Kunming 650500, China; 3 School of Electronic Science and Engineering, University of Electronic Science and Technology, Chengdu 611731, China; 4 Tianjin Key Laboratory of Optical Thin Films, Tianjin Jinhang Technical Physics Institute, Tianjin 300308, China

**Keywords:** single crystal diamond, dielectric properties, growth regulation, mechanism, stress, THz windows

## Abstract

The single-crystal diamond (SCD) possessing both favorable dielectric properties and low stress is esteemed as the ideal material for terahertz windows. The intrinsic step-like growth pattern of SCD can easily lead to stress concentration and a decrease in dielectric performance. In this study, a “two-step method” was designed to optimize the growth mode of SCD. A novel large platform growth pattern has been achieved by controlling diamond seed crystal etching and the epitaxial layer growth process. The experimental results indicate that, compared with the traditional step-like growth model, the root mean square (RMS) roughness of as-prepared SCD reduced from 5 nanometers (step growth) to 0.4~1.0 nanometers (platform growth) within a 5 μm × 5 μm area. Furthermore, the growth step height difference diminished from 30 nm to 3~4 nm, thereby mitigating stress induced by steps to a mere 0.1976 GPa. Additionally, at frequencies ranging from 0.1 to 3 THz, the diamond windows exhibit lower refractive index, dielectric constant, and dielectric loss. Finally, large platform growth effectively reduces phenomena such as dislocation pile-up brought about by step growth, achieving low-damage ultra-precision machining of diamond windows measuring 1 mm in diameter.

## 1. Introduction

The THZ wave is situated within the transition region from macroscopic electronics to microscopic photonics in the electromagnetic spectrum. This region serves as the boundary between macroscopic classical theory and microscopic quantum theory, exhibiting vast potential in areas such as national defense, military industry, radar detection, and biomedicine, among others [1,2,3,4]. In recent years, the rapid advancement of THz technology across various fields has necessitated the demand for high power and high efficiency transmission from THz sources [5]. The THz traveling wave tube (TWT) windows, as typical integrated structural–functional devices, are subject to constraints imposed by their dimensional architecture, necessitating the achievement of high quality and performance within a minimal size [6,7,8]. Consequently, the impact of size in the higher frequency bands significantly restricts the available options for manufacturing materials for windows.

Diamond materials possess a characteristic face-centered cubic structure, which exhibits the most tightly packed arrangement of carbon atoms. This unique crystal structure imparts diamond materials with distinctive attributes, including high mechanical strength (Vickers hardness of ~100–150 GPa, compressive strength exceeding 60 GPa), thermal properties (room-temperature thermal conductivity of ~2000–2200 W/(m·K) for natural diamond, thermal expansion coefficient of ~1.1–1.5 × 10^−6^ K^−1^), optical properties (spectral transmission range spanning from ~225 nm in the ultraviolet to beyond 25 μm in the far-infrared), and electrical properties (dielectric constant as low as 5.7 at room temperature, dielectric loss typically below 10^−4^ in the terahertz frequency range), among others [9,10,11]. As a result, numerous scientists have affirmed that the diamond is regarded as an ideal material for developing high-frequency THZ TWT windows. For instance, Ding and Pasagadagula et al. have reported progress in fabricating multilayer composite diamond films by utilizing ultra-nanocrystalline diamond (UNCD), traditional polycrystalline diamond (PCD), and microcrystalline diamond (MCD). These composite films show high mechanical strength and effective sealing performance, thereby making them highly suitable for implementation in THZ frequency band devices [12,13,14,15,16]. CPI company [17] employed a 1.4 mm-diameter, 0.076 mm-thick single crystal diamond as the RF window for TWT. The device achieves an output power of over 50 W at 220 GHz, with a power-bandwidth product surpassing 1000 W-GHz. Furthermore, in order to expand the application areas, Pasagadagula et al. [14] utilized CVD diamond as the dielectric material window, achieving a remarkably high operating frequency of 693 GHz accompanied by a return loss of less than 20 dB. Su et al. [18,19] devised a novel diamond energy transmission windows sheet by employing a two-stage waveguide window. The simulated windows sheet possesses a thickness of 0.1 mm, ensuring enhanced structural strength and vacuum compared to the cylindrical waveguide. Additionally, the operating band can reach 220 GHz. The attenuation and voltage standing wave ratio (VSWR) were also thoroughly analyzed by modifications of the thickness and diameter of the windows’ pans. The results demonstrate that the diamond window yields an attenuation of approximately −0.2 dB, and the VSWR remains below 1.1 within the frequency range of 220 GHz to 250 GHz. This performance aligns perfectly with the practical requirements for 0.22 THz TWT. So far, research on diamond windows has mainly focused on the design of polycrystalline diamond window structures and the optimization of different types of diamond windows in various wavelength bands. Research on optimizing and controlling the growth patterns of single-crystal diamonds for enhancing the performance of THz window slices is limited.

It is widely known that single crystal diamonds exhibit a step-like growth pattern during their growth process. These steps mainly originate from defects and impurities present on the seed surface [20]. Subsequently, carbon atoms laterally deposit on these steps, promoting the expansion and growth of SCD. However, the step flow growth mode easily leads to the formation of epitaxial layers with rough surfaces and step-induced stress. This has a detrimental impact on the manufacturing of high-quality THz window devices [21,22]. On one hand, single crystal diamond exhibits a step-like growth mode, while the epitaxial layer presents a step cluster situation, leading to an increase in intrinsic roughness. Diffusion scattering on the rough surface causes the absorption baseline to rise, thereby increasing dielectric losses. On the other hand, stress concentration occurs on these steps, making them prone to cracking and edge fractures during subsequent precision machining processes. Therefore, the key to solving the issue lies in enhancing the dielectric performance of materials and reducing stress by regulating the single crystal diamond step-like growth mode.

So far, in the realm of single crystal diamond growth regulation, studies have mainly focused on optimizing the diamond growth process and surface morphology treatment [22,23,24,25], with limited reports on the influence and control of growth patterns on dielectric properties. Dielectric performance is a crucial indicator for evaluating the performance of a power transmission window. Single crystal diamond’s favorable dielectric performance ensures that the power transmission window does not cause additional transmission losses when transmitting electromagnetic signals, thereby enhancing transmission efficiency. However, defects, non-smooth growth surfaces, high dislocation density, and the presence of a graphite (sp^2^ hybridization) phase during the growth process of single crystal diamond inevitably lead to an increase in dielectric constant and dielectric loss. Starting from the growth mode of single crystal diamond, it is essential to regulate it by optimizing diamond quality, surface roughness, and reducing stress to develop diamond window materials with low dielectric loss and low damage, which is key to addressing the issue.

In this paper, we start from the single crystal diamond step-like growth mode, successfully achieving the regulation of the “large platform growth effect” regulation by means of two steps: seed crystal etching and epitaxial layer control. Furthermore, we comprehensively analyze the differences in dielectric performance, stress, and other properties of SCD between the traditional step-like growth mode and the large platform growth mode, elucidating the relevant mechanisms. Finally, we have successfully prepared an SCD with a favorable dielectric performance and low stress while also achieving precise machining of small 1 mm-diameter windows with minimal damage. This provides a promising direction for the development of diamond materials for terahertz window applications.

## 2. Experimental Section

### 2.1. Diamond Deposition Growth

The substrate employed in the experiment was diamond seeds arranged along the 100 orientation, with dimensions measuring 3.5 mm × 3.5 mm × 1 mm. Prior to the growth or etching process, the aforementioned diamond seed was immersed in a piranha solution (H_2_SO_4_:H_2_O_2_ = 7:3) for 12 h at 80 °C to eliminate surface impurities and contaminants. Subsequently, it underwent ultrasonic cleaning with deionized water, alcohol, and acetone, respectively. Following this, the substrate was dried using N_2_ and placed within the deposition chamber. Further information regarding this deposition system has been described elsewhere [26].

The following etching and growth parameters can be found in Table 1. The E1 and E2 substrates represent epitaxial layers with or without pretreatment, while the G1, G2, and G3 substrates consist of epitaxial layers with varying methane concentrations. Throughout the etching process, the gas used for etching was H_2_ (at a flow rate of 400 sccm), the pressure within the reaction chamber was maintained at 8 kPa, the etching temperature ranged from 700–800 °C, the etching duration was set to 30 min, and the microwave power applied was 2000 W. During the growth process, precursor gases used were H_2_ and CH_4_. Adjustments to the pressure and microwave power were made to achieve a deposition temperature of 1000 °C for SCD. In our experimental arrangement, meticulous regulation was implemented on the CH_4_/H_2_ ratio (maintained at 2–4%) to govern the surface morphology of the epitaxial layer and inhibit step bunching.

### 2.2. Characterization

**Figure 1:**
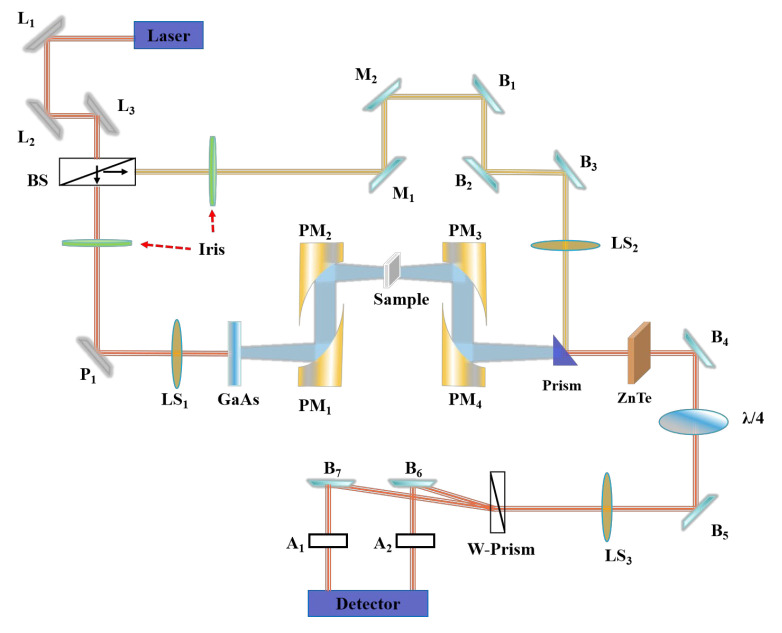
The schematic diagram of a THz-TDS system.

Scanning electron microscopy (SEM, Regulus 8230, Hitachi, Tokyo, Japan) was employed to characterize the morphology of the diamond surface, with an acceleration voltage set to 5 kV. X-ray diffraction (XRD, D8 DISCOVER, Bruker, Ettlingen, Germany) was utilized to analyze the crystalline orientation of the SCDs, using a Cu Kα target (wavelength λ = 0.154060 nm), and XRD scans were conducted in continuous mode over a 2θ range of 59.5–60°, with a step size of 0.002° and a step duration of 0.1 s. The surface roughness of the diamond was determined using an atomic force microscope (AFM, Dimension Icon, Bruker, Billerica, MA, USA)—a type of scanning probe microscope (SPM), the model of the cantilever beam is RTESSPA-150. Raman spectroscopy measurements were carried out using a Raman spectrometer (LabRAM Odyssey, Horiba, Lyon, France), with an excitation laser wavelength of 532 nm Spectral resolution: Full visible spectrum ≤ 0.65 cm^−1^, Full near-infrared spectrum ≤ 0.23 cm^−1^, Full ultraviolet spectrum ≤ 1.6 cm^−1^. The thickness of the diamond was measured using a metallographic microscope (Axio Observer 5, Zeiss, Oberkochen, Germany). Moreover, the dielectric properties of the as-prepared diamond were examined using THz time-domain spectrometry (THz-TDS), as shown in Figure 1. The THz-TDS system, provided by the Yangtze Delta Institute of Electronic Science and Technology University, has an experimental measurement range from 0.1 to 5 THz. The system consists of various components such as the L, P, and B, which are planar reflectors, the M, which is a delayed reflector, and the LS, A, BS, λ/4, PM, and W-prism, representing the focusing lens, attenuator, beam splitter, quarter-wave plate, off-axis parabolic mirror, and Wollaston prism, respectively. The terahertz waves undergo a series of reflections and interactions before illuminating the surface of the SCD, collecting the reflected and transmitted information and subsequently analyzing the dielectric properties of the SCD.

## 3. Results and Discussion

### 3.1. The Growth Regulation of SCD by “Two-Step Method”

**Figure 2:**
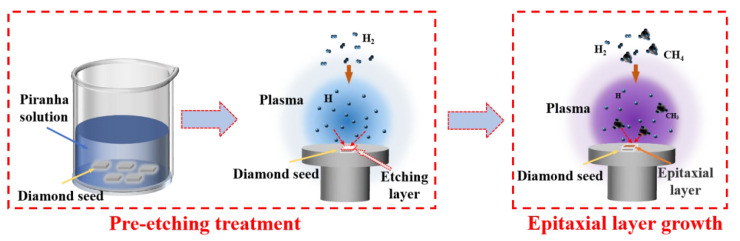
Schematic of the experimental process of “Two-Step Method”.

The intrinsic morphology of diamond seed crystal wafers exerts a profound influence on the epitaxial growth layer. To address this, we developed an optimized “Two-Step Method” for synthesizing single-crystal diamond with enhanced dielectric properties and lower stress. This will be achieved by intricate regulation of diamond substrate processing and exfoliation layer growth refinement. The experimental procedure of this method is illustrated in Figure 2. Firstly, we refine the morphology of the substrate by plasma etching, specifically employing hydrogen plasma to selectively erode the graphite phase and eliminate impurity defects. This step ensures that the substrate surface is meticulously cleansed and controlled, thus creating a conducive environment for subsequent deposition. Secondly, we exercise precise control over the growth pattern of the epitaxial layer by modulating the methane concentration. By the manipulation of the CH_4_/H_2_ ratio, we are able to steer the growth pattern of the epitaxial layer, leading to its deposition in an almost ideal fashion. Consequently, we successfully obtain an epitaxial layer featuring substantial uniformity and minimal roughness.

#### 3.1.1. First Step: Optimization of Single Crystal Diamond Seed

**Figure 3:**
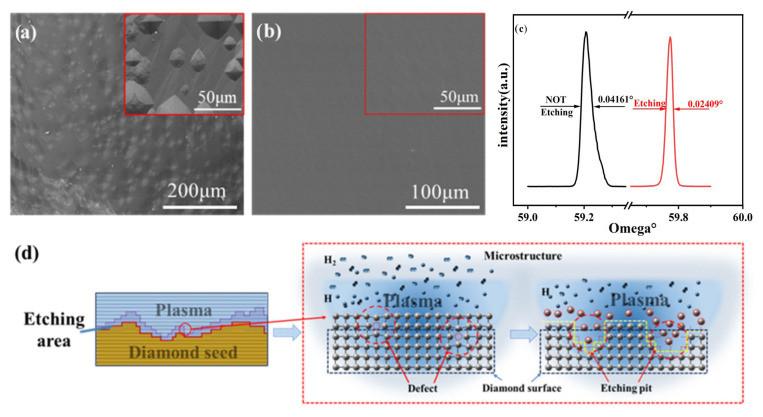
SEM images of epitaxial layers (**a**) without pretreatment and (**b**) with pretreatment; (**c**) XRD double rocking curve (ω-2θ) results (**d**) The corresponding etch mechanism.

To elucidate the underlying etching mechanism, we meticulously investigated the impact of pre-etching on the morphology of the epitaxial layer under preparation. Figure 3a and Figure 3b depict the surface topography of the deposited epitaxial layer with and without etching treatment, respectively. We have observed that the surface of the epitaxial layer lacking pre-etching treatment displays unevenness, characterized by distinct steps and the presence of multiple “Pyramids”. This phenomenon arises due to residues of impurities and dust on the seed crystal surface, as well as hidden defects (such as point defects and line defects), which serve as nucleation sites. Consequently, during the deposition process, these points of nucleation take precedence, leading to the growth of “Pyramid” islands. Significantly, this can also be regarded as the “Origin” of layer growth. Conversely, the surface of the epitaxial layer grown after pre-etching treatment appears flat, devoid of noticeable step bunching and “Pyramid” island growth. The implications of pre-etching treatment on the quality of the SCD grown in the epitaxial layer can be further demonstrated by the change in the full width at half maximum (FWHM) of the XRD results between the two conditions. Specifically, the FWHM for untreated growth measures 0.04161°, whereas for pretreated growth it is reduced to 0.02409°, as seen in Figure 3c. Figure 3d illustrates the mechanism of plasma pre-etching treatment. The primary purpose of hydrogen etching treatment is to remove organic impurities, dust, graphite phase, and surface defects such as point vacancies on the seed crystal. The elimination of diamond at an atomic level primarily involves the removal of atoms, leading to the stripping of surface C atoms and their combination with H atoms, thereby generating CH groups. This process enables precise control of surface morphology and defect removal, ultimately achieving successful surface modification. These experiments convincingly demonstrate that pre-plasma pre-etching treatment effectively regulates the subsequent epitaxial layer growth mode. By mitigating the formation of “pyramid” island-like growth and minimizing the prominent step bunching phenomenon, the growth pattern can be transformed into an approximate layer-like structure. Consequently, the quality of the epitaxial layer is substantially enhanced. In conclusion, employing pre-etching treatment prior to growth significantly contributes to the successful cultivation of high cleanliness and superior quality large platform SCD crystals.

#### 3.1.2. Step Two: Regulation of External Layer Growth

**Figure 4:**
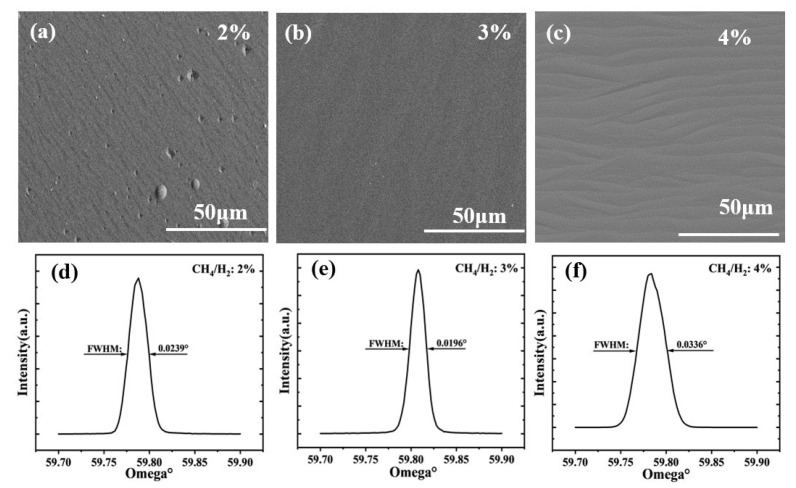
SEM morphology and corresponding XRD (ω-2θ) findings of epitaxial coatings with varying ratios of CH_4_/H_2_. (**a**,**d**) CH_4_/H_2_ = 2%; (**b**,**e**) CH_4_/H_2_ = 3%; (**c**,**f**) CH_4_/H_2_ = 4%.

**Figure 5:**
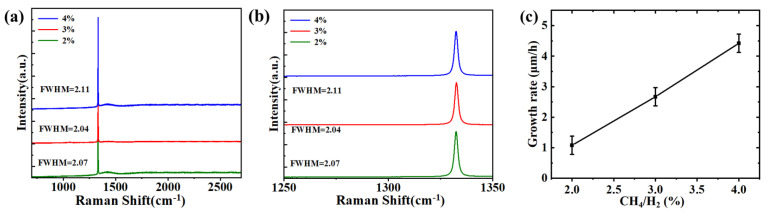
(**a**) Raman spectra under different CH_4_/H_2_ ratios; (**b**) Enlarged Raman spectra in the wavenumber range of 1250–1350 cm^−1^; (**c**) Measuring the growth rate of SCD epitaxial layers under different CH_4_/H_2_ ratios using a metallographic microscope.

In Section 3.1.1, we achieved seed crystals with enhanced surface quality. The subsequent step entails regulating the epitaxial layer of high quality, which is achieved by modifying the ratio of CH_4_ to H_2_. Figure 4 shows the SCD grown on the substrate morphology and XRD plots for varying CH_4_/H_2_ ratios (that is G1, G2 and G3 in Table 2, respectively). From the illustration, it is evident that the growth pattern of SCD undergoes significant changes as the CH_4_/H_2_ ratio fluctuates. Figure 4a shows that the deposition rate of SCD is lower than the etching rate of diamond by hydrogen plasma because of the low concentration. There are some circular protrusions on the surface of the epitaxial layer, which may be caused by the presence of dust and impurities selectively formed at this position. Figure 4c shows the cluster morphology with a stepped structure, which shows that when the CH_4_/H_2_ ratio is too high, the vertical growth rate exceeds the horizontal growth rate. In the step growth process, the growth process is accompanied by a slight hydrogen plasma etching effect in the cavity. The surface of CVD deposit in Figure 4b is flat, which proves that CVD diamond with wide flatness can be obtained by controlling methane concentration. Based on the findings depicted in Figure 4d–f, it can be observed that the FWHM values of the dual rocking curves for the respective SCDs are 0.0239°, 0.0196°, and 0.0336°. The FWHM of the XRD rocking curve is a key indicator of crystalline quality; a smaller value signifies a lower density of defects, such as dislocations, and therefore higher crystal quality. This outcome further confirms the significant influence of CH_4_ concentration on the quality of the epitaxial layer [27]. Figure 5 illustrates the Raman spectra of the epitaxial layer at various concentrations and depicts the correlation between the CH_4_/H_2_ ratio and the growth rate. The Raman plots reveal a degradation in the quality of the epitaxial layer (with an intensified fluorescence background) as the methane concentration increases. The FWHM value increases from 2.04 cm^−1^ at the optimal CH_4_/H_2_ ratio of 3% to 2.11 cm^−1^. Similarly, the narrower the full width at half maximum (FWHM) value of the Raman spectrum, the higher the quality of the grown diamonds, consistent with the conclusions drawn from X-ray diffraction (XRD) FWHM analysis [28]. Thus, we can deduce that the pretreatment process, in conjunction with precise control over the CH_4_/H_2_ ratio, is pivotal in achieving high-quality, large-scale, and highly pristine SCD growth.

**Figure 6:**
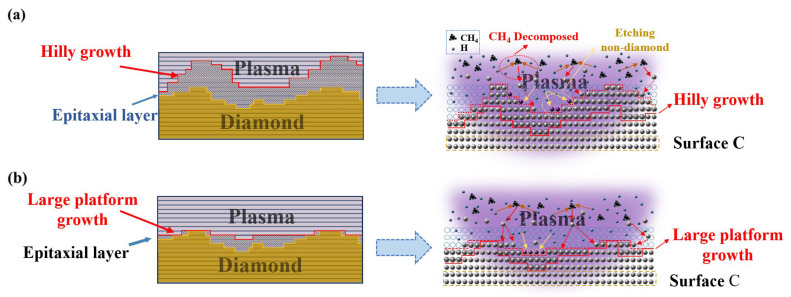
The corresponding growth mechanism of different growth modes of SCD. (**a**) The growth mechanism of the growth mode characterized by mound-like and step-like structures; (**b**) The growth mechanism of the large platform growth mode.

In the growth of CVD SCD, methane (CH_4_) serves as the exclusive carbon source. Upon entering the cavity and encountering hydrogen plasma, CH_4_ gas produces methyl groups, which subsequently react with H atoms to form carbon-containing groups denoted by C_x_H_y_. These C_x_H_y_ groups then settle onto the surface of the seed crystals and engage with the surface carbon atoms, ultimately giving rise to a gas-solid hybrid interface on the seed crystals’ surface. After undergoing a series of physicochemical reactions, the development of diamond phase (sp^3^), graphite phase, or amorphous carbon (sp^2^) is eventually achieved. Concurrently, the hydrogen plasma present within the cavity selectively erodes the graphite phase and amorphous carbon (sp^2^), while the diamond phase (sp^3^) undergoes a slow etching, thereby facilitating the growth of diamond. Figure 6 depicts the mechanism of the two distinct growth modes observed in SCD epitaxial layers. Figure 6a shows the growth patterns characterized by mound-like and step-like structures, where the vertical deposition rate (V_y_) of the diamond phase surpasses the horizontal rate (V_x_). Consequently, this growth mode exhibits increased film thickness, an augmented surface roughness, and a decline in diamond quality. Figure 6b depicts the growth mechanism of the large platform, where carbon atoms first fill the “vacancies” in the step grooves before growing layer by layer. This growth mode excels in greatly reducing the step height, controlling it at the atomic scale, and manifesting as a decrease in surface roughness at the macroscopic level. Simultaneously, the plasma energy has ample time to etch the accompanying graphite phase, effectively enhanced the quality of single-crystal diamond growth.

### 3.2. Comparison of Performance of SCD Prepared Under Two DIFFERENT Growth Patterns

#### 3.2.1. Effect of SCD Growth Patterns on the Dielectric Properties in the THz Band

**Figure 7:**
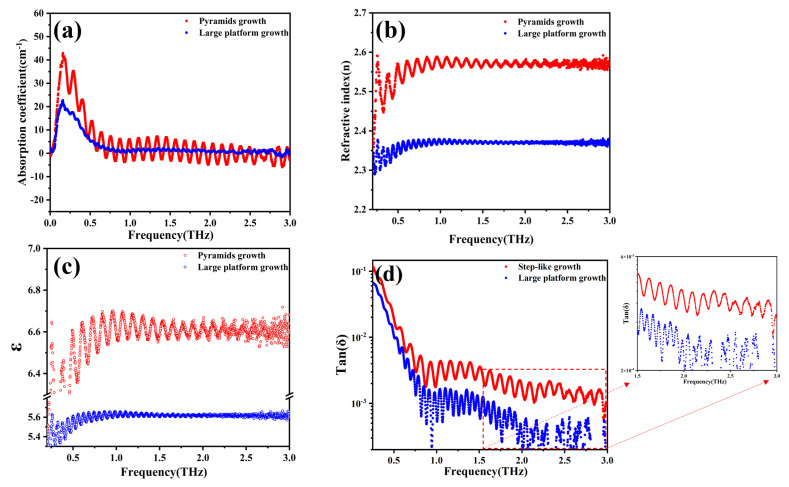
Effect of growth patterns on the dielectric properties of THz (The curve corresponding to ‘Step-like growth’ is drawn in red, and the curve corresponding to ‘Large platform growth’ is drawn in blue.). (**a**) Absorption coefficient; (**b**) Refractive index; (**c**) Permittivity; (**d**) Dielectric loss.

From the preceding section, it is evident that changes in growth patterns can have a significant impact on the surface morphology of the extension layer. The surfaces of SCDs grown in step-like patterns exhibit step clustering phenomena and even pyramid-like growth, which can have deleterious effects on surface roughness and quality. As shown in Figure 7a, dielectric performance testing was conducted on SCDs grown under two different growth patterns. It was observed that SCDs grown in step-like patterns showed stronger absorption peaks near 0.2 THz, with significant noise fluctuations across the entire 0.1–3.0 THz detection range. The cause of this phenomenon may be attributed to the α-C vibrations within the step-like patterns grown on the substrates. Meanwhile, the noise fluctuations can be explained by the Fabry-Pérot (FP) interference effect: part of the THz wave is reflected at the “air–upper SCD surface” interface, while another part transmits through the SCD and is reflected again at the “lower SCD surface–air (or SCD–substrate)” interface. The two reflected THz wave components (from the upper and lower surfaces) have a path difference determined by the SCD thickness and refractive index. As the test frequency changes, this path difference varies relative to the THz wavelength, leading to alternating constructive interference (which amplifies the signal) and destructive interference (which weakens the signal) between the two reflected waves. Furthermore, Figure 7b indicates a noticeable increase in the refractive index n value for SCDs grown in step-like patterns. This is attributed to the step clustering during step-like growth, resulting in increased surface roughness and a raised absorption baseline. The relationship between calculated dielectric constants and dielectric performance, refractive index, and extinction coefficient is depicted by Equations (1)–(4). Among them are n: refractive index, κ:extinction coefficient, ε′: real part of the complex permittivity (dielectric constant), ε″: imaginary part of the complex permittivity, and α: absorption coefficient.

(1) $$\;\varepsilon \;\prime \prime =\frac{\alpha \cdot \lambda ο}{2\pi \cdot n}$$

(2) $$\varepsilon \left(\omega \right)=\;\varepsilon \;\prime \left(\omega \right)-i\;\varepsilon \;\prime \prime \left(\omega \right)$$

(3) $$\;\varepsilon \;\prime \left(\omega \right)=n^{2}\left(\omega \right)-\kappa^{2}\left(\omega \right)$$

(4) $$\;\varepsilon \;\prime \prime \left(\omega \right)=2n\left(\omega \right)\cdot \kappa \left(\omega \right)$$

In the terahertz frequency range, there is an approximate relationship n = $\sqrt{\varepsilon }$ for obtaining the complex dielectric constant of materials. Therefore, a higher n value corresponds to a larger ε value in step growth, which has been confirmed by experimental evidence as shown in Figure 7c. Correspondingly, there is a noticeable increase in dielectric loss and noise phenomena in the step growth pattern, as depicted in Figure 7d. According to experimental results, it was found that by adjusting the growth pattern (from step growth pattern to large platform growth pattern), the dielectric constant values of the SCDs across the 0.1–3 THz range decreased from 6.6 to 5.6. The dielectric loss tangent values all showed a decreasing trend, in the 2–3 THz range, and the magnitudes of the large platform-grown SCDs are several times lower than those of the step-like growth SCDs. This is smaller compared with the values reported in [29,30,31], indicating an enhancement in its dielectric properties. It should be supplemented that these morphological optimizations have an inherent synergistic relationship with the reduction of sp^2^-bonded carbon content and the reduction in defect density, and the three together contribute to enhancing dielectric properties. Specifically, the reduction of surface roughness directly reduces the diffuse reflection scattering of THz waves, while the removal of sp^2^-bonded carbon on the seed surface by hydrogen plasma pre-etching and the suppression of defect aggregation by uniform layer-by-layer growth eliminate THz absorption centers from the perspectives of chemical composition and crystal substrate. The synergy of the two realizes the significant optimization of dielectric properties, which can be further verified by the characterization results of Raman spectroscopy in Figure 5 (no characteristic peak of sp^2^ carbon, FWHM = 2.04 cm^−1^) and XRD in Figure 4 (FWHM of double rocking curve = 0.0196°).

**Figure 8:**
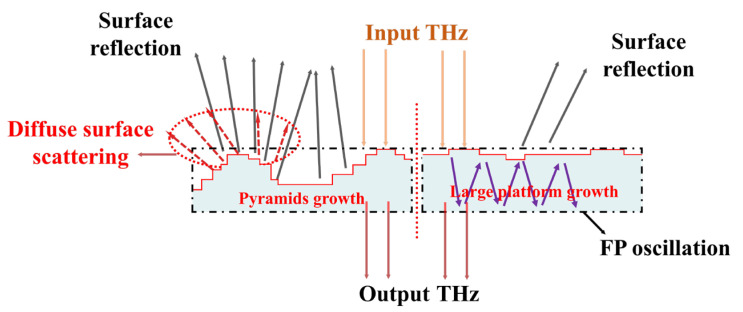
The influence mechanism of growth patterns on THz waves.

In Figure 8, a schematic diagram illustrating the scattering and absorption of THz waves under different growth modes is shown. Step-like growth patterns can significantly increase the roughness of the SCD surface, leading to the occurrence of diffuse scattering phenomena that elevate the absorption baseline of the SCD, while also causing a noticeable increase in the SCD’s refractive index. FP oscillation is an inevitable recurring phenomenon in the detection process of SCDs, manifested by the magnitude of detection noise. Conversely, SCDs prepared under the large platform growth mode can effectively avoid the generation of surface diffuse scattering phenomena, resulting in substantial boosts in the dielectric performance of the diamonds.

#### 3.2.2. Effect of SCD Growth Patterns on Stress

**Figure 9:**
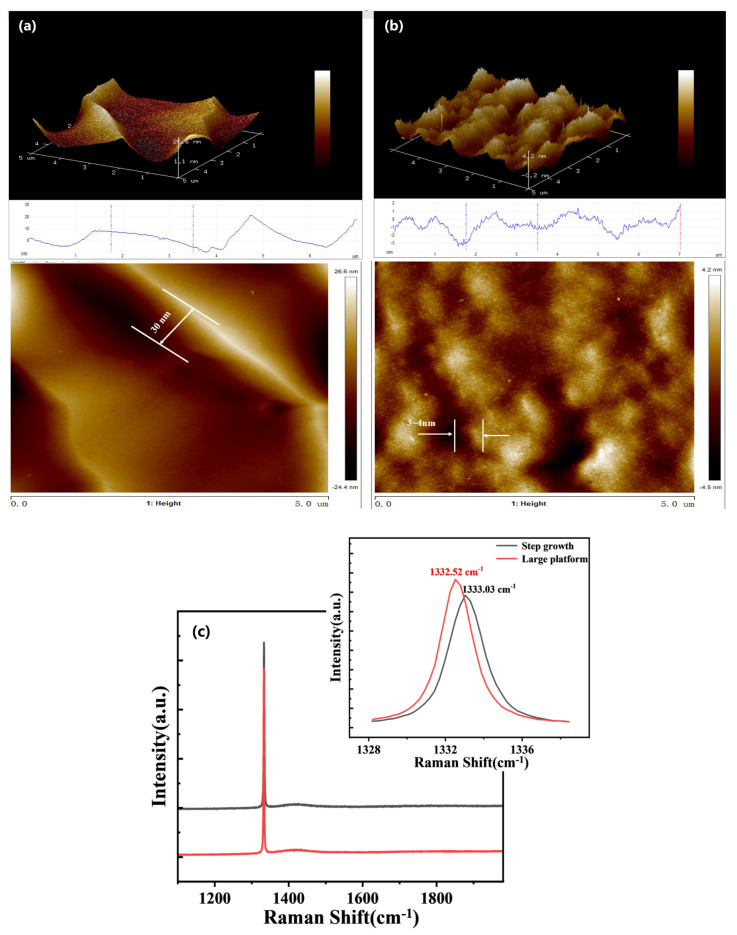
AFM maps of different growth modes of as-prepared SCD. (**a**) Step-growth mode; (**b**) Large platform growth mode; (**c**) The corresponding Raman spectra.

The growth mechanism of SCD primarily relies on the step mode, wherein the resulting continuous step surface is prone to step clustering phenomena. Furthermore, step growth inherently entails height disparities, thereby introducing novel step-induced stress on the surface which, in turn, exerts detrimental effects on subsequent precision processing endeavors. The objective of this experiment is to minimize the disparity in step height during step growth by adjusting the growth parameters, so as to convert the step-like growth into a more uniform large platform growth. As depicted in Figure 9, by regulating the growth parameter (CH_4_/H_2_ ratio), we managed to transition the growth mode from significant step growth to a large platform growth. The original step height of 30 nm was reduced to a mere 3–4 nm, resulting in a ten-fold reduction. This decrease in step height difference also led to a decrease in the stresses caused by the presence of steps. The corresponding surface stress of the epitaxial layer can be computed using Equation (5) [32].

σ = (V_R_ − V_R0_) × 0.38 GPa/cm^−1^ (5)

V_R_ represents the veritable epitaxial layer Raman characteristic peak, whereas V_R0_ symbolizes the standard diamond Raman feature peak (V_R0_ = 1332 cm^−1^). Consequently, with this knowledge, we can deduce that the stress of the prominent step amounts to 0.3914 GPa, as based on the diamond Raman feature peak shown in Figure 9c. By altering the growth pattern, we succeeded in mitigating the clustering phenomenon and the variance in step height, thus resulting in the root mean square (RMS) value of the epitaxial layer roughness plummeting from 5 nm to a range of 0.4–1.0 nm, leading to a reduction in surface stress upon the epitaxial layer to a mere 0.1976 GPa. Therefore, it is irrefutable that we possess the method to manipulate the mode of epitaxial layer growth by controlling the CH_4_/H_2_ ratio. This, in turn, diminishes surface strains, engenders enhanced quality in singular crystals, and offers advantages for subsequent precision processing.

**Figure 10:**
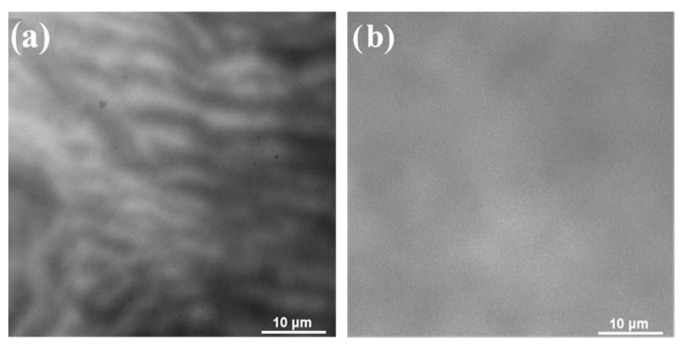
The CL plots of the different growth patterns. (**a**) Step growth mode; (**b**) Large platform growth mode.

In order to more intuitively observe the distribution of surface defects in different growth modes, characterization using CL imaging (Cathodoluminescence Imaging) is employed, as shown in Figure 10. Figure 10a,b represent step-flow growth and large platform growth, respectively. It is clearly visible that step-like growth exhibits dark shadows gathering at the steps, indicating a high density of defects accumulated at these locations. On the other hand, large platform growth shows a low and uniform surface defect density, leading to an equally distributed stress. This further confirms the significant optimization of stress and defect distribution by means of surface regulation.

**Figure 11:**
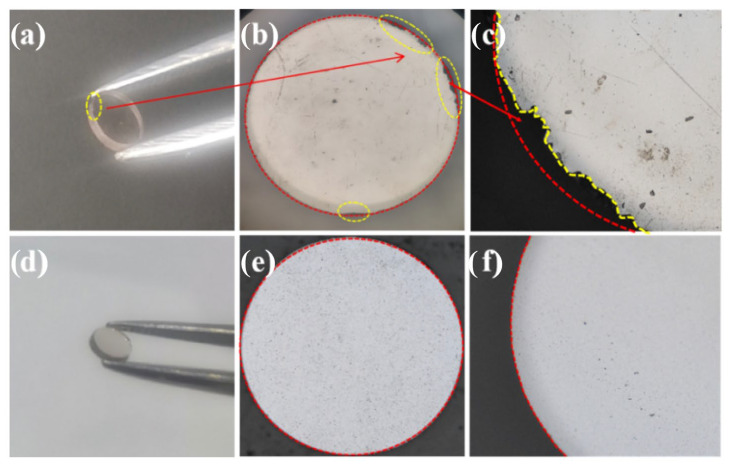
The optical microscope views of SCDs with different growth patterns after laser cutting are shown. (**a**–**c**) represent the step-like growth at 1×, 10×, and 50× magnification, while (**d**–**f**) illustrate the large platform growth at 1×, 10×, and 50× magnification.

The as-prepared SCD materials will ultimately be intended for the fabrication of THz windows. Consequently, laser ultra-precision machining becomes an indispensable process for the production of these small-sized (The diameter measures 1 mm, while the thickness stands at 200 μm.) windows. However, the traditional growth mode engenders significant step-related stress, rendering the edges susceptible to chipping cracks during laser processing, as depicted in Figure 11a–c. This experiment effectively mitigates the height of the growth step as well as the accompanying stress produced during the deposition process by utilizing the “two-step method” for fabricating the SCD windows. Therefore, during the processing, surface and sub-surface damages are effectively avoided, and the generation of cracks at the edges is suppressed, as depicted in Figure 11d–f.

## 4. Conclusions

In this paper, a “two-step methodology” was employed to meticulously manipulate the surface morphology and growth patterns of the SCD epitaxial layer. The initial stage involves the utilization of H plasma etching, enhancing the overall cleanliness of the seed crystal surface, while effectively avoiding any surface defects or impurities caused during the deposition process. The next step entails artfully manipulating the ratio of CH_4_/H_2_ to ingeniously modulate the growth pattern. The disparity in height between successive growth steps was diminished to a mere 3–4 nm, with the root mean square (RMS) value detected by atomic force microscopy (AFM) plummeting from 5 nm to a range of 0.4–1.0 nm. And the stress is reduced from 0.3914 GPa to 0.1976 GPa. The large platform growth effect has markedly mitigated the occurrence of dislocation pile-ups stemming from step growth, leading to specimens manifesting diminished refractive index, dielectric constant, and dielectric loss within the frequency band of 0.1–3 THz. This advancement is greatly beneficial for the application of single-crystal diamond in the THz window. Meanwhile, the diamond materials prepared in this study exhibit a noticeable inclination towards minimal damage during the ultra-precise cutting and processing procedures in the small-sized THZ windows.

## Figures and Tables

**Table 1:** Etching and growth parameters.

Substrate	Pretreatment	Growth Parameter		
		H_2_/sccm	CH_4_/sccm	Other Conditions
E1	-----	400	12	Power: 3.8 kW Pressure: 16 kPa T: 1000 ± 10 °C Time: 12 h
E2	H_2_: 400 sccm Power: 2 kW Pressure: 8 kPa Temperature: 700–800 °C Time: 30 min			
G1			8	
G2			12	
G3			16	

**Table 2:** Variation in diamond thickness measured using a metallographic microscope.

	G1	G2	G3
Pre-depositional thickness	498.37 µm	499.70 µm	509.23 µm
Thickness after deposition	511.33 µm	531.74 µm	562.27 µm
Growth thickness	12.96 µm	32.04 µm	53.04 µm

## Data Availability

The original contributions presented in this study are included in the article. Further inquiries can be directed to the corresponding authors.
